# Framework for a Research-Based and Interdisciplinary Use of Sensors in Elementary Teacher Education

**DOI:** 10.3390/s24175482

**Published:** 2024-08-24

**Authors:** Maria João Silva, Margarida Rodrigues, Tiago Tempera

**Affiliations:** CI&DEI, Escola Superior de Educação de Lisboa, Instituto Politécnico de Lisboa, Campus de Benfica do IPL, 1549-003 Lisboa, Portugal; margaridar@eselx.ipl.pt (M.R.); tiagot@eselx.ipl.pt (T.T.)

**Keywords:** sensors, teacher education, roles of sensors, framework

## Abstract

Sensors should be integrated into teacher education, as they are essential tools in the digital practices needed for full participation in society. Electronic sensors can be used as laboratory/scientific tools, as everyday mobile learning tools, and as epistemic mediators in several scientific fields, as well as in interdisciplinary approaches. In this way, electronic sensors can play multiple roles in the main dimensions of teacher education. The aim of the research presented in this paper was to create a framework for the research-based and interdisciplinary use of sensors in elementary teacher education, based on the thematic analysis of seven case studies implemented in Portugal. The thematic categories used in the cross-case analysis were fundamental in revealing the different roles played by sensors in the different phases of the didactic sequences of the cases. Subsequently, the thematic analysis made it possible to identify patterns of affordances of sensors and to relate the multiple roles of electronic sensors to different areas of the Portuguese elementary teacher education model. The research synthesis made it possible to outline the framework perspectives. The resulting framework systematized and highlighted the affordances of sensors in pre-service and in-service elementary teacher education as scientific, epistemic, interdisciplinary, and didactic mediators. These affordances were revealed to be particularly important in data-driven inquiry problem-solving, pedagogical content knowledge, and professional knowledge development. The framework created can be expanded in future related research.

## 1. Introduction

Electronic sensors are fundamental tools in most everyday mobile technological devices. Specifically, smartphones and tablets integrate a set of sensors, because micro-electro-mechanical systems technology has enabled the miniaturization of location, motion, and environmental sensors [1]. For example, smartphones are equipped with GPS sensors, accelerometers, magnetometers, barometers, microphones, and cameras [1]. Moreover, in the last two decades, mobile, affordable, and robust plug-and-play sensors for environmental sensing have been made available and used in student-centered teaching activities [2,3].

While digital technologies are part of children’s everyday lives, the role of schools in teaching digital competencies is equally essential [4]. Specifically, schools provide a structured, equitable, and comprehensive education that goes beyond casual use and equips students with the critical thinking, ethical understanding, and diverse skills necessary to navigate and thrive in the digital world [5,6]. For instance, at home, children may use digital technologies primarily for gaming, social media, or entertainment. In different circumstances, at school, they can be exposed to a broader range of digital tools and applications used in diverse fields such as science, math, arts, and the humanities. This will broaden their understanding and appreciation of how digital technologies can be used creatively and effectively across disciplines. By ensuring that all children receive a well-rounded digital education, schools will help prepare the next generation for the challenges and opportunities of an increasingly digital future [7].

Incorporating sensors into teacher education can further contribute to enriching teachers’ future educational practices [8], making it possible to: place students in real-world contexts where they collaboratively solve problems with active engagement, while developing a sense of community and shared responsibility [9]; involve students in intentionally using sensors to collect information that is meaningful to their learning goals [10]; and involve students in decision-making based on the data they collect with sensors, thereby enhancing critical social skills and emotional intelligence [3]. Moreover, in teacher education, the didactic use of sensors can develop critical thinking skills by encouraging the critical use of information [11,12]. Beyond how to read and use data effectively, the didactic use of sensors means approaching data by asking: “What data is being collected? How is the data collected? What is the data supposed to represent? What is the data used for?” [13].

In elementary education, the use of sensors holds noteworthy value [14], particularly because sensors can be used by pre- and in-service teachers to scaffold children’s learning, by starting with concrete, hands-on activities and gradually moving to abstract representations [15,16]. Additionally, sensors help develop multisensory approaches that allow children to experience and interact with phenomena that combine sensory experiences with cognitive processes [17].

Digital practices, with mobile or fixed devices, are mostly mediated by electronic sensors and are essential for full participation in society [18]. To prepare future citizens, education has been integrating digital technologies in recent decades, and this integration is specifically transforming teacher education [19]. However, there is still a long way to go to achieve effective global use of digital technologies and electronic sensors in teacher education [19].

The research presented in this paper aims to design a framework to support the research-based and interdisciplinary use of electronic sensors in elementary teacher education. To achieve this goal, it is necessary to address the following research question. What relationships between the roles of sensors and areas of elementary teacher education should be addressed in a framework to support the research-based and interdisciplinary use of electronic sensors in teacher education?

This framework’s design is based on the analysis of seven case studies of teacher education developed in Portugal. The set of selected case studies represents practices of sensor use in primary teacher education, previously developed and validated in the context of research projects such as the Eco-Sensors4Health project [20]. These practices have been implemented with pre-service and in-service teachers, in courses of fundamental areas of teacher education: pedagogical training (the scientific content of the subjects); specific didactics; and professional practice.

The analyzed case studies and related practices were framed by the European research-based, reflexive, critical, and social constructivist model of teacher education, which emphasizes the relational dimension and the interdisciplinary didactic reconstruction of content [21]. In this model, both teachers and teacher educators invest in practice-oriented and research-based professional development to construct innovative, reflective, and learner-centered teaching [15,21]. Accordingly, the use of sensors in teacher education should include the following dimensions: use of mobile sensors (controlled by students); didactic use; digital responsibility; culture, society, and democracy; development and transformation; and implications for epistemic practices [22].

To effectively integrate the abovementioned dimensions, the use of sensors in the analyzed teacher education case studies encompassed practices that combine:

- Students’ intentionality and agency in using sensors to acquire diverse data, based on learning the affordances of sensors [16];
- Didactic strategies that include problem-based collaborative situated learning [23,24], with collaborative learning of context-specific knowledge; concreteness fading [25], which scaffolds processes from concrete actions and observations to abstract representations; and embodied strategies [21], with multisensory approaches, using sensors as extended human senses [15];
- Students’ collaborative participation in decision-making based on data collected with sensors, enhancing emotional and social development in learning contexts [26];
- Inquiry strategies that promote epistemic practices, i.e., practices that construct knowledge and are similar to the practices of scientists, such as describing, predicting, using sensors, interpreting, organizing information, relating and making evidence-based decisions, and creating local knowledge [17].

The structure of this paper is organized as follows. It begins with this introduction, with the next section integrating the analysis of the potential role of sensors in elementary teacher education. The third section includes a description of the research design. The following two sections describe and discuss the research findings. Finally, the last section outlines the framework to support the research-based and interdisciplinary use of electronic sensors in elementary teacher education and is followed by a list of references.

## 2. Roles of Electronic Sensors in Elementary Teacher Education

### 2.1. The Portuguese Model of Elementary Teacher Education

Since the launch of the Lisbon Strategy in 2000, there has been a reform of policies related to teacher education, in which it began to be recognized as an academic field that contributes to improving the quality of teaching and the consequent impact on student learning [27]. As a result, the Bologna Process, adopted by various European Union countries, has had a significant impact on the restructuring of teacher education programs, promoting the renewal of the profession itself and the preparation of future teachers. Specifically in Portugal, the implementation of the Bologna Process in 2007 brought about significant changes in the conceptualization of teacher education, leading to a notable conceptual shift in the way teaching and learning are perceived [28].

Currently, teacher education in higher education institutions in Portugal follows the model prescribed by Decree-Law No. 43/2007 [29], under the joint responsibility of the Ministry of Education and the Ministry of Science, Technology, and Higher Education. This model includes guidelines first for the scientific training and then for the didactic training of students, with curricula for teacher training in basic education divided into six areas: General Pedagogical Training; Training in the Teaching Field (subjects’ scientific content); Specific Didactics; Cultural, Social, and Ethical Training; Training in Educational Research Methods; and Initiation to Professional Practice.

In accordance with Portuguese legislation, to become an elementary school teacher, students must complete a three-year broad degree in basic education, consisting mainly of theoretical and practical courses, followed by a two-year master’s degree in teaching, which includes a strong didactic component and professional practice corresponding to supervised teaching practice.

### 2.2. Electronic Sensors as Mainstream “Things”

The use of sensors has become ubiquitous in our daily lives, merging the physical and digital worlds and transforming human interaction with the environment [30]. Everyday objects, whether fixed, such as faucets, doors, or automobiles, or mobile, such as mobile digital devices (e.g., smartphones and tablets), sense various processes and physical properties [30], such as light, location, and motion. Sensors in smartphones are used pervasively, in an embodied way, and sometimes as part of personal identity by children, adolescents, and adults [31].

Data from sensors in homes, buildings, vehicles, and the environment are used to create a digital representation of the world [32]. Sensors are fundamental to the “things” dimension and paradigm of the Internet of Things (IoT) [33], and smartphones can be used as interfaces in this ecosystem [34].

Today, sensors have become mainstream tools used daily to support various aspects of life, such as [32,34]: (i) safety, e.g., the multiple sensors of cars or the sensor-controlled burglary and fire prevention systems at home; (ii) conservation, e.g., faucet sensors or light sensors; (iii) automation of daily activities, e.g., the environmental sensors for watering plants and heating in homes or the motion and distance sensors in doors; (iv) fitness, e.g., distance, motion, or location sensors; (v) health monitoring, e.g., the various sensors used to measure body variables.

Given that most of today’s individual and social activities use electronic sensors and the Internet, electronic sensors need to be explicitly addressed in formal education and consequently in teacher education. In line with IoT systems, in this research, sensors are used in elementary teacher education to collect data that are sent to be registered and analyzed [34].

While the IoT typically refers to an automated connection between sensors and objects’ actuators [8], this research takes a complementary approach, focusing on the use of mobile sensors by students—elementary schoolchildren and pre- and in-service teachers—as tools for data collection and use within their learning practices. This research strengthens the intentional/planned use of mobile sensors by students, and the link between sensor data and actions is not automated, being the result of data-based collaborative student decisions in problem-solving activities. Importantly, the focus of this research is not on programming and robotics but on data-driven collaborative sense-making.

### 2.3. Electronic Sensors as Tools in Technological and Scientific Practices

Formal education systems should ensure that all children and youth have access to science and technology education that focuses not only on technological and scientific products but also on scientific practices and processes, as well as on emotions, attitudes, and values [35,36].

Scientific practices—ways of doing, thinking, and talking in science—are essential components of science education [37]. These practices include the following [38]:

- Asking questions
- Developing and using models
- Planning and carrying out investigations
- Analyzing and interpreting data
- Using mathematics and computational thinking
- Constructing explanations
- Engaging in arguments from evidence
- Obtaining, evaluating, and communicating information.

To embed scientific practices in students’ worldviews, real-world contexts, and problems, the development of scientific practices in science education cannot be separated from engineering and mathematical practices [37,38]. Therefore, in science, technology, engineering, and mathematics (STEM) education approaches, the aforementioned scientific practices are intertwined with other practices [39]: asking questions (science) with defining problems (engineering); using mathematics and computational thinking (science) with using mathematics and information and computer technology (engineering); constructing explanations (science) with designing solutions (engineering).

In this context, sensors are fundamental tools for collecting environmental and laboratory data. Alongside sensors, data loggers in smartphones, tablets, or laptops also facilitate data storage and visualization in multiple representations [40] and support data handling, analysis, and interpretation.

Given the abovementioned affordances, physical and chemical sensors should be ubiquitous in laboratory classes to enable the detection and quantification of properties and processes [36]. Similarly, environmental sensors are fundamental tools in natural science fieldwork to monitor and assess environmental quality. In investigations that use scientific practices, sensors complement human senses and promote confrontation with students’ predictions, interpretations, and data-based explanations [36,37].

Scientific practices are fundamental in elementary education [40] and in elementary teacher education, since pre- and in-service teachers, as learners, should experience authentic science practices and should be scaffolded in how to put them into practice with their future pupils [40,41]. To understand and outline the relationship of the scientific role of sensors with the scientific, didactical, and practice areas of teacher education, this research analyzes the aforementioned scientific affordances of sensors in the diverse case studies’ practices.

### 2.4. Electronic Sensors as Epistemic Mediators

Science, technology, and engineering practices have an epistemic dimension because knowledge and learning are produced [42]. In carrying out these practices, in the context of inquiry, students are active epistemic agents who develop knowledge in practice in a collaborative and participatory way [43]. Here, knowledge and learning are produced, not just transmitted, as cognitive authority is distributed across the classroom community, emphasizing evidence-seeking, clarification, argumentation, and the sharing of ideas [43].

To aid in this process, epistemic mediators serve as external artifacts that function as a “prosthesis for the mind, expanding its possibilities and suggesting new information worth analyzing” [15]. These mediators are engaged through intentional, manipulative, and embodied actions [15].

Mobile electronic sensors can be used as epistemic mediators in educational science, technology, and engineering practices, such as making and interpreting observations, identifying problems, communicating in work teams, and evaluating the implications of data-based solutions [44]. When these practices are guided by problem questions and planned to generate scientific/technological/engineering investigations, students as epistemic agents can use sensors to focus on relevant aspects of phenomena in their autonomous sense-making operations [15].

In this way, students can use sensors with awareness of the environmental information that can be obtained through these tools [45], control the sensed data, and search for consistent information [46]. Additionally, they can collaboratively visualize and interpret data to make informed decisions and evaluate their consequences. Therefore, as epistemic mediators, sensors can scaffold students’ epistemic agency.

However, for teachers to foster epistemic agency in their professional practices, they must first experience being epistemic agents in their own formal education [47]. This research, therefore, analyzes the epistemic affordances of sensors in the practices of the selected case studies, aiming to frame the potential of the epistemic role of sensors in the scientific, didactic, and practical areas of teacher education.

### 2.5. Electronic Sensors as Didactic Tools

In teacher education, to promote the didactic use of sensors in their future practice, it is important that pre-service and in-service teachers experience the use of electronic sensors in the context of diverse didactic experiences. The affordances of electronic sensors, such as smallness [32], integration in mobile devices [1], and ease of manipulation [48], facilitate the use of mobile electronic sensors in self-determined, learner-driven, and collaborative learning strategies [49], such as inquiry- and problem-based learning.

Inquiry- and problem-based learning require the posing of questions and scientific/technological/engineering practices based on empirical data, either by directly manipulating variables in experimental work or by constructing comparisons using data sets [50]. Therefore, in the research process, there are complex processes that require metacognitive skills and conceptual and procedural knowledge, namely processes of constructing meaning, which involve interpreting data, and strategic control decisions, with reconstruction, evaluation, and articulation of previous knowledge [50].

Furthermore, in inquiry- and problem-based learning, sensors can be used as didactic tools because they can: (i) be manipulated by students in a deliberate and planned manner [46]; (ii) scaffold students in collecting empirical data (physical, chemical, and environmental) needed to answer the initial problem questions [48]; and (iii) facilitate collaboration [49] by providing interfaces with laptop and tablet applications to easily create and share multiple representations of the acquired data. As a result, while using sensors in inquiry- and problem-based learning, students experience authentic learning and produce local (situated) knowledge to solve real-world problems in their own contexts [51].

Moreover, when specifically used as extensions of the human senses, sensors support teachers in helping students bridge sensory information to more abstract representations, such as graphical representations of sensor data, in a didactic approach called concreteness fading [25].

To evidence the sensors’ potential as didactic tools in the scientific, didactical, and practice areas of teacher education, it is relevant to analyze the aforementioned didactic affordances of sensors in the practices of the selected case studies. This analysis is implemented in the present research.

### 2.6. Electronic Sensors as Interdisciplinary Tools

In a complex and challenging world, students need to draw on interdisciplinary knowledge to understand the multifaceted and interconnected issues they face [52]. An approach based on fragmented knowledge linked to isolated subjects does not help students to develop a deeper understanding of the different phenomena they are studying. Thus, a relevant curriculum implies making connections between subjects [53].

Building on this idea, it is important to promote in teacher education the development of interdisciplinary pedagogical content knowledge (IPCK) [54] for pre-service and in-service teachers to achieve an interdisciplinary approach, which involves the use of common concepts to bring together disciplinary subjects without separating disciplinary knowledge and skills [55]. IPCK is the ability to use themes or shared concepts across curricular boundaries, addressing content areas from multiple subjects simultaneously.

For example, when pre-service and in-service teachers address a topic across curricular boundaries [54], such as in the context of identifying authentic environmental problems, sensors can be used as interdisciplinary tools to collect data that are then organized, represented, and interpreted to answer the questions posed. In this scenario, this type of work involves addressing content from multiple disciplines simultaneously and recognizing common concepts and processes [55], including concepts such as proportion (e.g., analyzing and making sense of the variation in relative humidity measured with sensors), quantity (estimating, analyzing, and making sense of the variation in sound level as a function of site characteristics and human behavior), cause and effect (e.g., the effect of plants on the concentration of carbon dioxide in the air), and the stability and change of systems.

For the above-mentioned reasons, it is important for pre-service and in-service teachers to be able to design lessons that focus on making connections within and across subjects. By doing so, the curriculum becomes more relevant by placing multiple subjects in context and linking them to the real world [53]. In the learning process, students “generate, consolidate, and share meanings by making connections between signs, objects, and their application in real-world contexts” [56]. Specifically, sensors, along with data loggers, provide representations that are technologically mediated and visual and mathematical in nature. Therefore, students develop their representational literacy when they can visualize, create, and analyze these representations as tools for reasoning and making claims. Moreover, in order to maintain an interdisciplinary approach, it is necessary to ensure the authenticity of the activity, which means that students focus on problem-solving outside the target disciplinary framework during the instructional process [56].

As Moreira et al. [57] point out in relation to open data and the Internet of Things, data collected by students using sensors are important resources for problem-solving, particularly in collaborative research-based activities. In these settings, students develop goals, collect data, organize and analyze, argue, and engage in other epistemic practices to solve and communicate real-world problems in an interdisciplinary manner.

In this research, the interdisciplinary sensors’ affordances are identified in the analysis of the case studies’ practices, whenever such affordances are made explicit by teachers’ educators to pre- and in-service teachers.

## 3. Research Design

The main goal of the present research is to design a framework to support the research-based and interdisciplinary use of electronic sensors in teacher education. It is important to evidence the main set of design options, adopted by the authors to attain this goal:

- A pragmatic worldview was recognized and assumed since the characteristics of the main goal require an educational problem-centered approach, which demands real-world practices focused on the consequences of actions [58].
- A qualitative research method was adopted to acknowledge the complexity of the goal, allow emerging questions and practices, and collect data in the participants’ settings [58].
- The analysis of case studies was the research option to support the framework design, since this framework should be situated, rely on multiple sources of evidence, and data collection and analysis should be guided by prior development of theoretical propositions [59].

Following those research options, the specific objectives were then designed to operationalize the framework design:

(i) Analyze a series of case studies on the use of sensors in teacher education in Portugal to identify the role of sensors in the different research-based activities.
(ii) Relate the role of sensors to the diverse areas of the Portuguese model of teacher education (General Pedagogical Training; Training in the Teaching Field (subjects’ scientific content); Specific Didactics; Cultural, Social, and Ethical Training; Initiation to Professional Practice).

The first step of this research was the definition of the set of case studies. The selection was based on the following criteria: (i) each case study describes and evaluates a didactic sequence on the use of sensors to produce learning outcomes; (ii) all case studies were subject to academic evaluation (academic peer review or jury); (iii) the set of case studies includes cases developed with pre-service and in-service teachers in the scientific, didactical, and practice areas of teacher education.

Another selection criterion, previously mentioned in the Introduction of this paper, is that every selected case must be based on the European research-based, reflexive, critical, and social constructivist model of teacher education, to allow analyzing the diverse roles of sensors in the case studies’ practices. Furthermore, the set of selected cases includes scientific inquiries on solving problems, such as climate change, but also ones that are considered the main environmental health problems in schools, such as noise, indoor air quality, and thermal comfort [60].

The selected set of case studies includes seven cases: (i) one case study with a didactic sequence on plants and indoor air quality, developed in the Living World course of the undergraduate degree; (ii) two case studies developed in the Digital Technologies in Mathematics and Science interdisciplinary course of the undergraduate degree, each with a different didactic sequence and a different topic (heart rate and location and noise pollution); (iii) one case study with a didactic sequence on thermal comfort problems in schools, developed by pre-service teachers and implemented with fifth-grade children, in the Mathematics in Environmental Issues course of the Master’s Degree in Teaching; (iv) two case studies, each developed in the final practicum of a pre-service teacher of the Master’s Degree in Teaching, with a different didactic sequence and a different topic/grade level (relationship between climate change and biodiversity/fifth-grade class and thermal comfort in schools/third-grade class); and (v) a case study with a didactic sequence on noise pollution in schools, developed by two pre-service teachers and implemented with first- and fourth-grade classes, in the context of a workshop.

To explore why, where, and how sensors were used in the case studies’ learning practices, an exploratory reading of these cases was carried out to identify and select the in-depth analysis categories. Didactic sequence phases, context, content, and strategies, the role of sensors, and learning outcomes were then defined as such categories. Those seven case studies were considered sufficient in number, not only because of the aforementioned criteria but also because supplementary case studies, which also followed the selection criteria, did not add new thematic issues, and the main concepts of the theory (see Section 2) are already addressed.

Each case study describes and evaluates a didactic sequence in which sensors are used as tools. “Context”, “Contents”, “Strategies”, and “Learning outcomes” are essential dimensions of didactic sequences and are required to understand the use of sensors in the sequences. Learning outcomes are the results of students’ scientific and epistemic practices and include the acquisition of conceptual and procedural knowledge, as well as competencies. In different “Didactic Sequence Phases”, there are different “Roles of sensors”, as characterized in Section 2 (Electronic Sensors as Laboratory/Scientific Tools, Epistemic Mediators, Didactic Tools, and Interdisciplinary Tools).

The second research stage was an in-depth analysis of the case studies to explore why, where, and how sensors were used in empirical research in real-world contexts [59]. This was a necessary step to enable the third stage—research synthesis. Research synthesis is used to synergistically create new interpretations and conclusions that go beyond the sum of the conclusions of the empirical studies analyzed [61]. The authors adopted a thematic analysis approach to identify patterns/themes in the data [61] and use a realist perspective in which the researchers present the previous assumptions and methods to be tested based on evidence, aiming to explain “what works for whom, in what circumstances, in what respects, and how” [62].

## 4. Results

In this section, a series of seven case studies on the use of sensors in teacher education are presented, analyzed across and thematically, and synthesized. These case studies were developed in the context of research projects in which the authors of this paper have been involved.

### 4.1. Case Studies in Basic Education Degree Courses

The first case study focuses on a didactic sequence aimed at learning about the influence of plants on indoor air quality in the *Living World* science course of the undergraduate degree [63]. In this didactic sequence, pre-service teachers study photosynthesis and respiration processes; learn how to use sensors of carbon dioxide in the air while measuring this variable in different air conditions; use these sensors in experimental activities on carbon dioxide exchange in photosynthesis and plant respiration; and collect, organize, process, and interpret sensor data to conclude on the influence of plants on air quality. In this first case, the carbon dioxide sensors were used by the teachers of the course as didactic tools to explore the affordances of sensors in the study of air quality, namely in terms of observation, searching for evidence, and the visualization of data (Table 1). Sensors were also used as laboratory tools to carry out experimental activity on carbon dioxide exchange in photosynthesis and plant respiration. Finally, sensors were used as epistemic tools to support pre-service teachers in organizing, processing, interpreting, and communicating data.

The next two cases focus on two didactic sequences of the *Digital Technologies in Mathematics and Science* course of the Basic Education degree, which is an interdisciplinary course. The didactic sequence of the second case is based on learning about heart rate in different contexts and activities (Table 2), such as exploring the outdoor spaces of the school, up and down the paths of the campus, with different levels of difficulty and safety. Heart rate sensors, together with Google Earth, allow the joint visualization of heart rate data, location, and elevation [64]. In the first phase of this didactic sequence, pre-service teachers, with teacher mediation, learn about heart rate in different people, contexts, and activities, and how to use heart rate sensors to study this diversity. In this way, pre-service teachers are asked to perform a series of tasks that include planning, collecting, organizing, processing, and analyzing heart rate data. In these tasks, sensors are used as didactic tools by teacher educators in the first phase of the didactic sequence. In the following phases, sensors are used by pre-service teachers as scientific tools in the scientific data-based activities, interdisciplinary tools, using mathematics and science tools and common concepts—such as cause-and-effect and spatial orientation and location—to make sense of heart rate data in diverse activities, and epistemic tools in knowledge creation and communication practices.

The third case study (Table 3) was developed in the same course but with a didactic sequence on sound problems in school. The global strategy of this didactic sequence is scientific inquiry [9]. Accordingly, pre-service teachers are challenged to use sound sensors to collaboratively identify sound problems and answer related problem questions in school. In this context, sound sensors are used as didactic tools by teacher educators to allow understanding and to apply the sound sensors’ affordances [9]. Furthermore, sensors are used as scientific tools to support the collection, organization, processing, and application of sound level data. They are also used as interdisciplinary tools, since they support the use of mathematics and natural sciences tools and shared concepts—such as proportion and quantity and cause-and-effect—to make sense of sound level data while developing scientific inquiry. This way, sound sensors are also used as epistemic tools in inquiry, knowledge creation, and communication practices.

**Table 1:** Characterization of a didactic sequence on plants and indoor air quality in the *Living World* course of the Basic Education degree [63].

Didactic Sequence Phases	Context	Contents	Strategies	Role of Sensors	Learning Outcomes
Conceptual exploration: photosynthesis and respiration Exploration of the Activity Guide	Pre-service teachers’ classroom activities	Photosynthesis; Respiration; Indoor air quality: pollutants and safety values	Group work		Knowledge of photosynthesis and respiration processes Knowledge of indoor air quality: pollutants and safety values
Exploratory use of sensors	Pre-service teachers’ classroom and outdoor curricular activities	Carbon dioxide sensors’ affordances	Group work	Didactic tools	Knowledge of carbon dioxide sensors’ affordances Competencies in the use of sensors
Laboratorial use of sensors	Pre-service teachers’ laboratory and curricular activities	Carbon dioxide in air: variables and measurements Photosynthesis, respiration, and Gas Exchange Multiple representations of environmental information Statistical measures	Laboratory group work	Laboratory/Scientific tools Epistemic tools	Sense of measurement of carbon dioxide in air Competencies in the use of multiple representations and statistical measures in data organization, processing, and interpretation
Reports’ development	Pre-service teachers’ autonomous curricular activities	Communication of environmental information and knowledge	Group work	Epistemic tools	Conceptual and procedural knowledge of the influence of plants on indoor air quality Communication and synthesis competencies

### 4.2. Case Study in Teacher Education Master’s Degree Interdisciplinary Course

The fourth case focuses on a didactic sequence aimed at identifying the problem of thermal comfort in school in the Mathematics in Environmental Issues course of the master’s degree in teaching (Table 4). Although this course is scientific, it has a didactic approach to facilitate the connection with the future practice of pre-service teachers. Thus, this didactic sequence was planned by a group of pre-service teachers and implemented in the Teacher Education Campus with a fifth-grade class [65]. The activities were carried out both indoors and outdoors. After a conceptual exploration of temperature, relative humidity, and thermal comfort, the students were organized into groups. Some groups measured temperature in different locations and the other groups measured relative humidity in different locations using sensors and a script with cues for spatial orientation. Each group of students plotted the collected data on a thermal comfort graph. Since this diagram is a Cartesian system, the students had to interpret the two scales presented in the diagram and put into practice the notion of a coordinate point as the junction of the two values of temperature and relative humidity. The use of the diagram was essential for an effective understanding of the concept of thermal comfort, which relates the two variables of temperature and relative humidity. Afterwards, the students discussed and shared the results obtained and concluded on the problem of thermal comfort on campus.

In this case, temperature and relative humidity sensors were used by pre-service teachers as didactic tools to support children in exploring the affordances of sensors in exploring thermal comfort problems, namely in terms of observing, searching for evidence, and visualizing data. Sensors were also used as interdisciplinary tools, allowing students to integrate math and science to learn common concepts such as proportion and quantity. As science tools, sensors complemented students’ sense of thermal comfort and promoted interpretation and data-based explanation. Finally, sensors were used as epistemic tools to support children’s data processing, interpretation, and communication.

**Table 2:** Characterization of a didactic sequence on heart rate and location in the *Digital technologies in Maths and Science* course of the Basic Education degree [66].

Didactic Sequence Phases	Context	Contents	Strategies	Role of Sensors	Learning Outcomes
Activity planning Exploratory use of heart rate sensor	Pre-service teachers’ classroom activities	Heart rate Heart rate in different contexts and activities Heart rate sensors’ affordances	Group work	Didactic tools	Knowledge of heart rate in different contexts and activities Knowledge of heart rate sensors’ affordances in the context of Mathematics and Natural Sciences collaborative activities Competencies in the use of sensors
Experimental activities: data collection, processing, and interpretation	Pre-service teachers’ classroom and outdoor curricular activities Teacher mediation	Heart rate and location variables and measurements Multiple representations of heart rate and location information Statistical measures Methods and techniques of data organization and processing	Fieldwork: data collection Use of Excel in data processing	Scientific tools Epistemic tools Interdisciplinary tools	Knowledge of heart rate in different contexts and activities Sense of number and measurement of heart rate and location Competencies in the use of multiple representations and statistical measures in data organization and processing Competencies in the use of spreadsheets in the context of health and environmental tasks.
Reports’ development	Pre-service teachers in autonomous work	Communication of health and environmental information and knowledge	Communication in the activity process	Epistemic tools	Communication and synthesis competencies

**Table 3:** Characterization of a didactic sequence on sound pollution in school in the *Digital technologies in Maths and Science* course of the Basic Education degree [9].

Didactic Sequence Phases	Context	Contents	Strategies	Role of Sensors	Learning Outcomes
Exploration of the Activity Guide Inquiry planning	Pre-service teachers’ classroom activities	Sound level Sound problems in schools	Group work		Knowledge of sound level and sound problems in schools Inquiry planning competencies
Interdisciplinary use of sensors: exploratory activities	Pre-service teachers’ classroom and outdoor curricular activities	Sound sensors’ affordances	Practical work: use of sensors	Didactic tools	Knowledge of sound sensors’ affordances in the context of Mathematics and Natural Sciences collaborative environmental activities Competencies in the use of sensors
Inquiry activities: planning, data collection, processing, and interpretation	Pre-service teachers’ classroom and outdoor curricular activities Teacher mediation	Sound level variables and measurements in school Multiple representations of environmental information Statistical measures Methods and techniques of data organization and processing	Fieldwork: data collection Use of Excel in data processing	Interdisciplinary tools Scientific tools Epistemic tools	Sense of number and measurement of sound level Competencies in the use of multiple representations and statistical measures in data organization and processing Competencies in the use of spreadsheets in the context of environmental inquiry tasks
Inquiry reports: presentations and debate	Pre-service teachers in classroom activities	Communication of environmental information and knowledge	Communication in the inquiry process	Epistemic tools	Communication and synthesis competencies

**Table 4:** Characterization of a didactic sequence design on thermal comfort problems in school in the *Mathematics in Environmental Issues* course of the master’s degree in teaching [65].

Didactic Sequence Phases	Context	Contents	Strategies	Role of Sensors	Learning Outcomes
Conceptual exploration: temperature, relative humidity, and thermal comfort	Fifth-grade children, mediated by pre-service teachers during classroom activities	Temperature and Relative Humidity Thermal comfort problems in schools	Debate in whole class Individual registration of concepts Sharing of concepts Systematization of concepts		Knowledge of temperature, relative humidity, and thermal comfort problems in schools
Interdisciplinary use of sensors Inquiry activities: data collection	Fifth-grade children, mediated by pre-service teachers in classroom and outdoor curricular activities	Temperature and relative humidity sensors’ affordances	Practical work: use of sensors in several locations, following clues (spatial orientation) Fieldwork: data collection	Didactic tools Interdisciplinary tools	Knowledge of temperature and relative humidity sensors’ affordances in the context of Mathematics and Natural Sciences collaborative environmental activities Competencies in the use of sensors
Inquiry activities: data processing and interpretation	Fifth-grade children, mediated by pre-service teachers in classroom activities	Temperature and relative humidity variables and measurements in school Percentages Multiple representations of environmental information Statistical measures Coordinates	Group work: data recording in a thermal comfort diagram	Scientific tools Epistemic tools	Sense of number and measurement of temperature and relative humidity Competencies in the use of multiple representations and statistical measures in data processing Competencies in the use of coordinates in a Cartesian system.
Inquiry reports: presentations and debate	Fifth-grade children, mediated by pre-service teachers in classroom activities	Communication of environmental information and knowledge	Communication in the inquiry process Comparison of the results obtained by the groups	Epistemic tools	Communication and synthesis competencies

### 4.3. Case Studies in Internships of the Master’s Degree in Teaching

The fifth case study focuses on a didactic sequence aimed at linking climate change and biodiversity (Table 5). This case was part of a study conducted by a pre-service teacher in the final practicum of her master’s degree [67]. In this case, the pre-service teacher guided fifth-grade students to learn how to use sensors to measure carbon dioxide in the air and the pH of the water in eco-chambers under different conditions: before and after candle burning and before and after one of the students exhaled air. These experiments enabled the students to acquire knowledge about the sources of carbon dioxide in the air from human activities and the cascading consequences with negative impacts on biodiversity. The sensors were used as didactic tools, as part of the didactic sequence planned by the pre-service teacher, as laboratory/scientific tools for measuring air quality and water pH by the children, and as epistemic tools in knowledge creation, supporting data organization, results interpretation, and the communication of conclusions.

The sixth case integrates a didactic sequence aimed at improving elementary school students’ awareness of thermal comfort and problem-solving in their schools and contributing to the development of communication strategies to propose interventions to improve the school environment [68]. This case, like the previous one, was developed during the supervised teaching internship of a pre-service teacher. In the implementation of this didactic sequence (Table 6), third-grade students analyzed and identified the thermal comfort problems in their school with the help of sensors, measuring temperature and humidity in different locations. The experiment allowed the students to become aware of the thermal comfort problems in the school, and to reflect and create research-based solutions. Sensors were used as didactic tools in the experiment planned by the pre-service teacher, as scientific tools to measure air quality, and as epistemic tools to help organize data, design solutions based on the data obtained, and disseminate the solutions identified, promoting greater awareness of this issue.

**Table 5:** Characterization of a didactic sequence on the relationship between climate change and biodiversity in a pre-service teacher’s final internship for the master’s degree in teaching [67].

Didactic Sequence Phases	Context	Contents	Strategies	Role of Sensors	Learning Outcomes
Conceptual exploration: Climate change and biodiversity	Internship: fifth-grade classroom activity	Climate change Biodiversity	Debate, brainstorming		Identification of students’ prior knowledge about climate change and biodiversity
Experimental activity: Concentration of carbon dioxide on different air samples	Internship: fifth-grade classroom activity	Carbon dioxide concentration in air Air quality	Laboratory group work	Didactic tools Laboratory/Scientific tools Epistemic tools	Knowledge of carbon dioxide in air sensors’ affordances Competencies in the use of sensors Sense of measurement of carbon dioxide in air
Experimental activity: Measurement of carbon dioxide in air and water pH in eco-chambers before and after the combustion of a candle	Internship: fifth-grade classroom activity	Water pH Combustion	Laboratory group work	Didactic tools Laboratory/Scientific tools Epistemic tools	Knowledge of pH and carbon dioxide in air sensors’ affordances Competencies in the use of sensors Sense of measurement of pH in water and carbon dioxide in air
Experimental activity: Measurement of water pH in eco-chambers after exhaled air	Internship: fifth-grade classroom activity	Water pH Breathing	Laboratory group work	Didactic tools Laboratory/Scientific tools Epistemic tools	Knowledge of the affordances of pH sensors and carbon dioxide in air Competencies in the use of sensors Sense of measurement of pH in water and carbon dioxide in air
Systematization of the whole process and learning outcomes	Internship: fifth-grade classroom activity	Climate change Biodiversity	Debate Questionnaire	Epistemic tools	Communication Synthesis competencies Knowledge acquisition about climate change and biodiversity

**Table 6:** Characterization of a didactic sequence on a school’s thermal comfort during the internship of a pre-service teacher [68].

Didactic Sequence Phases	Context	Contents	Strategies	Role of Sensors	Learning Outcomes
Introduction to the temperature and relative humidity concepts. Thermal comfort multisensory exploration	Internship: third-grade classroom activity	Temperature and relative humidity	Group work		Basic knowledge and multisensory awareness of temperature and relative humidity
Data collection with sensors and interpretation and identification of problems in their school	Internship: third-grade outdoor and indoor activities	Thermal comfort, temperature, and relative humidity	Experimental work on thermal comfort in several locations	Didactic tools Scientific tools Epistemic tools	Knowledge of thermal comfort problems in school Knowledge of thermal comfort sensors’ affordances Competencies in the use of sensors Competencies in the use of multiple representations and statistical measures in data processing Competencies in the use of coordinates in a Cartesian system
Reflection on the problems found and possible improvement solutions	Internship: third-grade classroom activity	Thermal comfort, temperature, and relative humidity	Group work	Epistemic tools	Formulation of solutions to the identified thermal comfort problems in school
Sharing the improvements/solutions found	Internship: third-grade classroom activity	Thermal comfort, temperature, and relative humidity	Debate Communication	Epistemic tools	Communication and synthesis competencies Knowledge acquisition about thermal comfort

### 4.4. Case Studies in Teacher Education of In-Service Teachers

In the seventh case, the didactic sequence aimed at identifying and solving the problem of noise pollution in the school. It was developed in the teachers’ workshop “Create eco-healthy schools through the use of sensors by children” and promoted to teachers of first-to-fourth-grade and fifth- and sixth-grade mathematics and science [10]. Two in-service teachers from the same school jointly planned a didactic sequence for their classes (first and fourth grade), which had some common aspects and some specificities (Table 7). The children began by asking themselves what a sound is (first grade) and what it takes to hear the sounds and why we listen (fourth grade). The students explored a sound scale, which was a reference for classifying conditions as safe or risky for health. With this reference, the students made predictions (collectively in first grade and individually in fourth grade) about sound levels in the classroom, in the canteen, and in other places in the school. In this didactic sequence, children learn how to use sound sensors by measuring sound levels in different locations. They collect, process, and interpret the data from the sensors to compare with their initial predictions and draw conclusions about the problem of noise pollution in the school. They also proposed several solutions to the identified problems. In this case, sound level sensors were used by in-service teachers as didactic tools to explore the affordances of sensors in exploring sound problems, in terms of observation, searching for evidence, and visualizing data. Sensors were also used as interdisciplinary tools, facilitating interdisciplinary problem-solving in accordance with the math and environmental studies curricula. In this sense, sensors were also used as scientific tools to complement children’s senses and to promote evidence-based reflection on their predictions, as well as interpretation and explanation in data-based problem-solving. Finally, sensors were used as epistemic tools to support students’ data processing, interpretation, communication, and implementation of solutions.

**Table 7:** Characterization of a didactic sequence on sound pollution in school in the in-service teachers’ workshop, *Create eco-healthy schools, through the use of sensors by children* [10].

Didactic Sequence Phases	Context	Contents	Strategies	Role of Sensors	Learning Outcomes
Conceptual exploration: sound	First- and fourth-grade classroom activity Outdoor curricular activities	Sound	Debate in whole class Outdoor listening activity with distinction between pleasant and unpleasant sounds		Knowledge of sound and sound problems in schools
Formulation of predictions	First- and fourth-grade classroom activity	Sound level Sound problems in schools	Collective and individual predictions		Knowledge of sound level and sound problems in schools
Interdisciplinary use of sensors: exploratory activities Inquiry activities: data collection	Outdoor curricular activities	Sound level Sound sensors’ affordances	Group work Fieldwork: data collection	Didactic tools Interdisciplinary tools	Knowledge of sound level Competencies in the use of sensors
Inquiry activities: data processing and interpretation	First- and fourth-grade classroom activity, Teacher mediation	Sound level variables and measurements in school Multiple representations of environmental information Statistical measures	Data analysis Confrontation between the predictions and the measured values	Interdisciplinary tools Scientific tools Epistemic tools	Sense of number and measurement of sound level Competencies in the use and interpretation of multiple representations and statistical measures in data processing
Inquiry reports: presentations and debate Creation, dissemination, and implementation of solutions	First- and fourth-grade classroom activity, Teacher mediation	Communication of environmental information and knowledge Sound problem solutions in schools	Communication in the inquiry process Children’s agency in solutions and their implementation	Epistemic tools	Communication and synthesis competencies Competencies in citizen intervention

## 5. Discussion: The Roles of Environmental Sensors in Teacher Education

The results of the selected case studies make it possible to identify some affordances, as patterns of integrated potentialities, in the use of sensors in pre-service and in-service teacher education. The cross-case analysis revealed the different roles played by sensors in the different phases of the didactic sequences. A research synthesis is presented in the following two sections.

Due to the well-known capabilities of sensors to detect and measure environmental quantities in the real world [69], all the cases analyzed integrated didactic sequences that used scientific inquiry strategies to identify, explore, and solve real-world environmental problems. Depending on the different environmental problems, different environmental sensors were selected and used in the inquiries: sound sensors, temperature and humidity sensors, carbon dioxide sensors, pH sensors, and heart rate sensors. In all cases, students define the intentionality of the use of sensors, control them, and use the collected data for evidence-based analysis and decision-making.

In the first three case studies, the environmental investigations were designed and facilitated by teacher educators, the next three cases were designed and facilitated by preservice teachers, and the final case was designed and facilitated by in-service teachers. In this way, sensors were actively used by pre-service and in-service teachers, as well as elementary school students, at different stages of the case studies’ inquiry processes.

The first phase of all didactic sequences is aimed at preparing the following phases. Depending on the previous knowledge and skills of the students, the first phase included conceptual exploration (cases 1, 4, 5, 6, and 7) and/or multisensory tasks (cases 6 and 7) or even planning activities (cases 2 and 3).

In the following phases, sensors were used by the teachers who designed the activities as **didactic tools** or mediators that extended the students’ senses in the inquiry learning activities, such as data collection in all didactic sequences [48]. In case 7 (sound pollution, with first- and fourth-grade children), sensors were used by in-service teachers as didactic tools to scaffold children in improving their prediction skills while making sense of the sound level variable.

Case studies 1 (on the topic of plants and carbon dioxide, with undergraduate pre-service teachers), 2 (on the topic of heart rate, with undergraduate pre-service teachers), 5 (relationship between climate change and biodiversity, with fifth-grade children, mediated by a master pre-service teacher), and 6 (thermal comfort, with fifth-grade children, mediated by a master pre-service teacher) involved the use of sensors as **scientific tools** in experimental activities, as students developed practical work and controlled variables. In the other cases, the control of variables was not explicit, but sensors were also used as **scientific tools** to measure environmental variables [40].

In the seven didactic sequences of the case studies (Table 1, Table 2, Table 3, Table 4, Table 5, Table 6 and Table 7), environmental sensors were used as **epistemic tools**/mediators to scaffold the creation, validation, and communication of knowledge [44], during observation, and with data collection, organization, processing, and interpretation, as well as in the evidence-based formulation of problem solutions.

In the analyzed cases, the joint use of sensors and data loggers prioritized the use of tablets, as these mobile devices are efficient in facilitating collaborative work, collaborative learning, communication, inquiry-based learning, mobile learning, and multimodal learning [70]. Furthermore, the small size of smartphone screens does not facilitate children’s reading [71]. Nevertheless, in case studies 1, 2, and 3, some groups of pre-service teachers used their own smartphones as the **most important “things”** because they showed a preference for such interfaces.

Case studies 2 (on the topic of heart rate, developed with pre-service teachers in the *Digital Technologies in Mathematics and Science* course) and 3 (on the topic of sound pollution, developed with pre-service teachers in the *Digital Technologies in Mathematics and Science* course), as well as case 4 (thermal comfort, developed by pre-service teachers in the *Mathematics in Environmental Issues* course of the Master’s degree in teaching), were developed in interdisciplinary courses. Consequently, sensors were used as **interdisciplinary tools**, as pre-service teachers were guided by teacher educators to design and develop field work, making interdisciplinary strategies explicit. Data organization and process techniques, multiple representations, concepts, claim-making, and foundational learning common to mathematics and natural sciences are collected and applied in the context of scientific inquiry to solve complex and real-world problems [53,55]. Environmental sensors are interdisciplinary mediators that facilitate this collection and application. The other case studies also include the aforementioned interdisciplinary strategies. However, such strategies were not made explicit during the didactic sequences.

## 6. Conclusions: Framework for the Use of Sensors in the Diverse Areas of Teacher Education

In this section, the authors of this paper propose a set of statements as the first version of a case-situated framework for the use of sensors in different areas of teacher education. This first version can and should be expanded upon in future related research.

The case studies that were the basis of the research presented in this paper include didactic sequences implemented in the context of in-service and pre-service education. In pre-service education, the cases analyzed integrate didactic sequences in different areas: (i) disciplinary and interdisciplinary courses in the undergraduate program; (ii) an interdisciplinary course in the master’s program in teaching; and (iii) a final practicum in the master’s program in teaching.

In research-based teacher education, pre-service and in-service teachers should use research-based knowledge and experience and create research-related practice-oriented activities [72]. The research presented here reveals and illustrates the first statement of the framework. The use of sensors in scientific inquiry activities to solve real local environmental problems can be an important strategy in pre-service and in-service teacher education, as sensors provide opportunities for data collection, organization, processing, interpretation, and evidence-based proposals of solutions.

The seven case studies analyzed were developed in two undergraduate courses, one master’s course in teaching, the final practicum in the same master’s course, and an in-service workshop. In these contexts, pre-service and in-service teachers used sensors: (i) as scientific and epistemic tools to experience scientific inquiry in solving real local environmental problems and (ii) as didactic tools to design and implement inquiry didactic sequences with first- to fifth-grade classes, in which they mediated the use of sensors as scientific and epistemic tools by children.

The second framework statement can be formulated as follows. In pre and in-service teacher education, there is evidence of the affordances of sensors for use in research-based activities, not only as scientific and epistemic tools but also as didactic tools.

The case studies developed in the context of interdisciplinary courses demonstrated the role of sensors in supporting the focus on data-based authentic problem-solving, rather than on disciplines, as recommended by Tytler et al. [56]. However, the development of interdisciplinary teacher education requires not only overcoming disciplinary approaches but also making the links and connections between disciplines visible to pre-service and in-service teachers and making interdisciplinary intentionality explicit [53,55], as referred to in the previous section. Accordingly, the third framework statement can be expressed as: “Sensors can be used as interdisciplinary tools/mediators in pre-service and in-service teacher education, given their affordances as facilitators of data-driven problem-solving”. However, the interdisciplinary intention needs to be made explicit to develop IPCK and to achieve an interdisciplinary approach.

In the present research, the use of sensors as scientific, epistemic, and interdisciplinary tools, as well as didactic tools, by pre-service and in-service teachers in scientific inquiry activities to solve real local environmental problems revealed potentialities in different areas of initial teacher education, namely Training in the teaching field (scientific content of the subjects); specific didactics; and initiation to professional practice.

Cases 1 (plants and carbon dioxide, with undergraduate pre-service teachers), 2 (heart rate, with undergraduate pre-service teachers), and 3 (noise pollution, with undergraduate pre-service teachers) were developed in the context of in-service training (science content of subjects), with the aim of providing pre-service teachers with experience in using sensors to solve local environmental problems using a scientific inquiry strategy. Consequently, the fourth framework statement can be formulated as: Sensors can be used in teacher education to develop the scientific content of subjects and in the training of the teaching field, given their affordances as scientific, epistemic, and interdisciplinary tools.

Case study 4 (thermal comfort) was also developed in the context of a course on training in the teaching area (scientific content of subjects) (mathematics in environmental issues). However, in this course of the Masters in Teaching, the pre-service teachers, in addition to their own practice with sensors, also designed and implemented a didactic sequence with a fifth-grade class of a nearby school, in which the children used sensors to identify, explore, and solve local thermal comfort problems. The fifth framework statement is related to these specific pedagogical practices: Sensors can be used in teacher education to develop Pedagogical Content Knowledge, given their affordances as didactic tools.

Case Study 5 (relationship between climate change and biodiversity) was developed with a fifth-grade class, and the didactic sequence was designed and mediated by a Master Education pre-service teacher in his final internship. In addition, Case Study 6 (thermal comfort) was developed with a third-grade class, and the didactic sequence was designed and mediated by a Master Education pre-service teacher in her final internship. Therefore, it is possible to formulate the sixth framework statement: Sensors can be used by pre-service teachers in their final practicum (Initiation to Professional Practice area of initial teacher education), given their affordances as didactic, scientific, epistemic, and interdisciplinary tools.

The seventh and last case study (sound pollution theme) illustrates how sensors can be used by in-service teachers in their practice, from the first grade, with relevant learning outcomes, following the challenge of a teacher education workshop. In this way, the seventh and final statement of the framework reads: Sensors can be used in the continuing education of in-service teachers to improve and update their professional knowledge and competencies.

## Data Availability

Data are contained within the article.
